# Prenatal Household Air Pollution Alters Cord Blood Mononuclear Cell Mitochondrial DNA Copy Number: Sex-Specific Associations

**DOI:** 10.3390/ijerph16010026

**Published:** 2018-12-22

**Authors:** Seyram Kaali, Darby W. Jack, Rupert Delimini, Lisa Hu, Katrin Burkart, Jones Opoku-Mensah, Ashlinn Quinn, Kenneth Ayuurebobi Ae-Ngibise, Blair J. Wylie, Ellen Abrafi Boamah-Kaali, Steven Chillrud, Seth Owusu-Agyei, Patrick L. Kinney, Andrea A. Baccarelli, Kwaku Poku Asante, Alison Gladding Lee

**Affiliations:** 1 Kintampo Health Research Centre, Ghana Health Service, Brong Ahafo Region, P. O. Box 200, Kintampo Ghana; jones.opoku-mensah@kintampo-hrc.org (J.O.-M.); Kenneth.ae-ngibise@kintampo-hrc.org (K.A.A.-N.); ellen.boamah@kintampo-hrc.org (E.A.B.-K.); sowusuagyei@uhas.edu.gh (S.O.-A.); kwakupoku.asante@kintampo-hrc.org (K.P.A.); 2 Department of Environmental Health Sciences, Columbia University Mailman School of Public Health, New York, NY 10032, USA; dj2183@cumc.columbia.edu (D.J.); lh2736@cumc.columbia.edu (L.H.); katburk@uw.edu (K.B.); andrea.baccarelli@columbia.edu (A.A.B.); 3 Department of Biomedical Sciences, University of Health and Allied Sciences, P.O. Box 31, Ho, Ghana; rdelimini@uhas.edu.gh; 4 Fogarty International Center, National Institutes of Health, Bethesda, MD 20892, USA; ashlinn.quinn@nih.gov; 5 Division of Maternal–fetal Medicine, Department of Obstetrics and Gynecology, Beth Israel Deaconess Medical Center, Boston, MA 02215, USA; bwylie@bidmc.harvard.edu; 6 Lamont-Doherty Earth Observatory at Columbia University, Palisades, NY 10964, USA; chilli@ldeo.columbia.edu; 7 Institute of Health Research, University of Health and Allied Sciences, PMB 31, Ho, Ghana; 8 Department of Environmental Health, Boston University School of Public Health, Boston, MA 02118, USA; pkinney@bu.edu; 9 Division of Pulmonary, Critical Care and Sleep Medicine, Icahn School of Medicine at Mount Sinai, New York, NY 10029, USA

**Keywords:** household air pollution, oxidative stress, mitochondrial DNA copy number, sex-specific effects, fetal programming

## Abstract

Background: Associations between prenatal household air pollution (HAP) exposure or cookstove intervention to reduce HAP and cord blood mononuclear cell (CBMC) mitochondrial deoxyribonucleic acid copy number (mtDNAcn), an oxidative stress biomarker, are unknown. Materials and Methods: Pregnant women were recruited and randomized to one of two cookstove interventions, including a clean-burning liquefied petroleum gas (LPG) stove, or control. Prenatal HAP exposure was determined by serial, personal carbon monoxide (CO) measurements. CBMC mtDNAcn was measured by quantitative polymerase chain reaction. Multivariable linear regression determined associations between prenatal CO and cookstove arm on mtDNAcn. Associations between mtDNAcn and birth outcomes and effect modification by infant sex were explored. Results: LPG users had the lowest CO exposures (*p* = 0.02 by ANOVA). In boys only, average prenatal CO was inversely associated with mtDNAcn (β = -14.84, SE = 6.41, *p* = 0.03, per 1ppm increase in CO). When examined by study arm, LPG cookstove had the opposite effect in all children (LPG β = 19.34, SE = 9.72, *p* = 0.049), but especially boys (β = 30.65, SE = 14.46, *p* = 0.04), as compared to Control. Increased mtDNAcn was associated with improved birth outcomes. Conclusions: Increased prenatal HAP exposure reduces CBMC mtDNAcn, suggesting cumulative prenatal oxidative stress injury. An LPG stove intervention may reverse this effect. Boys appear most susceptible.

## 1. Introduction

Approximately 3 billion people worldwide are exposed daily to household air pollution (HAP) secondary to the burning of solid fuels in combustion-inefficient cookstoves [1]. HAP is a toxic mixture of pollutants including carbon monoxide (CO), nitrous oxides, particulate matter (PM), polyaromatic hydrocarbons (PAHs), aldehydes, and volatile organic compounds [2,3] and results in significant morbidity and mortality, particularly from acute and chronic respiratory and cardiovascular diseases [4,5,6]. All family members are exposed, however women are the predominant cooks and continue to cook while pregnant; thus, exposure to HAP begins in utero and continues across the lifecourse [7].

Alterations in fetal development, including those resulting from environmental exposures, may program the fetus for future disease [8,9]. Emerging evidence suggests that early life HAP exposure may explain a large proportion of the risks associated with HAP. Specifically, prenatal HAP exposure has been linked to adverse birth outcomes (i.e., low birth weight, stillbirth, and preterm delivery [10,11]), which may increase the risk of neonatal mortality [12], acute lower respiratory infection (ALRI) in childhood [13], and cardiorespiratory [14,15] disease later in life. Recent work by our group demonstrated that increased prenatal HAP exposure impaired infant lung function, which subsequently increased risk for ALRI in the first year of life [16]. The underlying mechanisms have not been elucidated, although oxidative stress pathways are believed central [17,18].

Environmental pollutants such as air pollution induce oxidative stress [17,19]. Human cells contain multiple mitochondria, each with many copies of mitochondrial DNA (mtDNA). Mitochondria are a main source of adenosine 5’-triphosphate (ATP) and central to cellular energy production. Mitochondria lack protective histones and have inefficient DNA repair systems making them susceptible to oxidative injury [20,21]. Thus, air pollution-associated oxidative stress may induce mitochondrial dysfunction via reduced mitochondrial DNA copy number (mtDNAcn). Previous work suggests that increases in prenatal ambient air pollution exposure is associated with reduced cord blood and placental tissue mtDNAcn [22,23]. Similarly, perinatal tobacco smoke exposure has been linked with mtDNA damage in blood and aortic tissue [24]. However, the effect of prenatal HAP exposure or a clean cookstove intervention to reduce HAP exposure on CBMC mtDNAcn has not been studied.

Fetal development requires the careful orchestration of complex processes across gestation and sex-specific differences in development have been demonstrated [25]. The impact of environmental exposures on mtDNAcn may have sex-specific differences. Rosa et al demonstrated that boys were especially susceptible to the effects of increased prenatal air pollution exposure on mtDNAcn [23]. Understanding sex-specific associations may help identify vulnerable populations.

Using a prospective, rural Ghanaian birth cohort derived from a cookstove intervention trial, we examined the impact of prenatal HAP exposure, as indexed by personal prenatal CO exposure assessments, and a cookstove intervention on CBMC mtDNAcn. Sex-specific differences were examined. Finally, we examined the associations between CBMC mtDNAcn and birth anthropometrics.

## 2. Materials and Methods

### 2.1. Study Participants

Participants were mother–infant dyads participating in the Ghana Randomized Air Pollution and Health Study (GRAPHS), a cluster randomized cookstove intervention trial described in detail elsewhere [26]. Briefly, between August 2013 and March 2016, *n* = 1414 pregnant, nonsmoking women were recruited from the Kintampo North Municipality and Kintampo South District of Ghana and randomized prior to the second trimester of pregnancy to a clean-burning stove, the liquefied petroleum gas (LPG) cookstoves; an improved combustion-efficiency biomass burning stove, the BioLite cookstove; or control, the traditional 3-Stone Fire. The analyses in this manuscript include *n* = 120 mother–infant dyads who delivered between October 2014 and August 2015 at gestational age > 37 weeks and had successful cord blood collection at delivery with subsequent CBMC mtDNAcn analyses.

The study was approved by the Kintampo Health Research Centre (KHRC) Institutional Ethics Committee (Federal Assurance Number 00011103, approval code: 2014-23/amendment 1) and the Institutional Review Boards of the Icahn School of Medicine at Mount Sinai and the Columbia University Mailman School of Public Health. Written informed consent was obtained from all pregnant women prior to commencement of study activities.

### 2.2. Prenatal CO Exposures

Pregnant women performed four 72-hour prenatal personal CO monitoring sessions (Lascar EL-CO-USB Data Logger, Essex, UK). Participants wore the personal monitor in their breathing zone except while sleeping or bathing, during which times they were asked to keep the monitor off the floor and nearby. A custom plastic case with a tea bag filter protected the CO monitor from rain and dust.

The CO monitors recorded readings in parts per million (ppm) every 10 seconds. Laboratory technicians exposed the monitors to certified span gas (50 ppm CO in zero air) every 6 weeks to quantify response and adjust field values. Additional visual inspection and run time checks of all exposure data were made following GRAPHS protocols. Data used in this analysis passed all Quality assurance and quality control checks.

CO exposure at each of the four prenatal sessions was based on the first 48 hours of each deployment, to avoid cases where field pick up schedules or battery issues could have resulted in incomplete data (i.e., a missed cooking session). The mean of each 48-hour personal CO monitoring session was averaged to determine the average prenatal CO exposure for each dyad. Forty-eight-hour data completeness for the GRAPHS cohort was high (93.6%); this was evenly distributed among intervention groups. 

### 2.3. Cord Blood Sampling and DNA Extraction

Immediately following delivery, cord blood was collected into BD vacutainer heparinized tubes and transported to the KHRC Clinical Laboratory on IsoRack cool packs (Iso Therm-System, Hamburg, Germany). Density gradient centrifugation (Ficoll-Paque^TM^) was performed to isolate CBMCs, which were immediately stored at −80 °C until analyses. DNA was extracted using the Qiagen DNA Midi Kit. DNA samples were normalized to 2 ng/µL and concentrations were confirmed using PicoGreen quantification prior to amplification. Samples failing to meet the minimum concentration were excluded from analysis.

### 2.4. MtDNAcn Measurement

MtDNAcn was assayed using duplex quantitative PCR (qPCR) to compare the relative amplification of nuclear and mitochondrial segments of DNA as previously described [27]. The primers for qPCR of mtDNA were mtF805 5’-CCACGGGAAACAGCAGTGATT-3’ and mtR927 5’-CTATTGACTTGGGTTAATCGTGTGA-3’. The RNaseP Copy Number Reference Assay (LifeTechnologies) was used to quantify nDNA. The PCR conditions were set up as follows 50 °C for 2 minutes, 95 °C for 10 minutes and 15 seconds, 60 °C for 1 minute, followed by 39 cycles of 95 °C for 15 seconds, and a melting curve at 65 °C. Each sample was run in triplicate and in addition, six random samples were run in duplicate from the normalization stage on each plate to ensure consistency of results. Each plate contained a standard curve as a reference. The standard curve was made from a tissue-specific pooled sample comprising equal mass of total DNA from all samples of that tissue type. Triplicate samples showed high reproducibility (Coefficient of Variation < 0.1). The calculated mtDNAcn, a percentage representing the sample’s mtDNAcn relative to the study average, was averaged across triplicates and used in subsequent analyses. Plate number was recorded.

### 2.5. Birth Outcomes

Birth anthropometrics were measured within 24 hours of delivery. Birth weight was measured to the nearest gram using the Tanita BD 585 digital baby scale (Tokyo, Japan) after standardizing with a 1-kilogram (kg) weight. Birth length and head circumference were also measured to the nearest 0.1 centimeter (cm) using a standard measuring board and a Lasso-o™ respectively. Gestational age at delivery was determined using ultrasound-established dates at GRAPHS enrollment; all ultrasounds were performed prior to 24 weeks gestation [28].

### 2.6. Covariates

Maternal age and ethnicity were obtained through questionnaires at enrollment. Maternal height to the nearest 0.1 cm and weight to the nearest 0.1 kg were measured at enrollment. Household assets were surveyed and used to assign a wealth index relative to others in the study. Date of birth was recorded at delivery and, in combination with the ultrasound-confirmed gestational age at delivery, used to calculate gestational age at delivery. Child sex was recorded at delivery.

### 2.7. Statistical Analysis

First, we employed multivariable linear regression models to determine the impact of prenatal HAP exposure, as indexed by CO and the GRAPHS cookstove intervention arm, independently, on CBMC mtDNAcn percentage relative to the study average. For analyses of cookstove intervention arm, the 3-Stone Fire was considered the referent. Models were adjusted for the following covariates, maternal height and weight, ethnicity, and age; household wealth index; gestational age at delivery; child sex; and plate number. Effect modification by child sex was then examined in stratified analyses and by fitting interaction terms. As the LPG stoves were considered the least polluting, we repeated analyses with the LPG arm as the referent.

We then employed multivariable linear regression models to determine associations between CBMC mtDNAcn and birth outcomes, including birth weight, head circumference, and gestational age at delivery, considered independently. We again adjusted for maternal height and weight, ethnicity, age; household wealth index; gestational age at delivery; child sex; and plate number. Effect modification by child sex was then examined in stratified analyses and by fitting interaction terms.

Main effects were considered statistically significant if the *p*-value was less than 0.05. Interaction terms were considered suggestive of an interaction if the *p*-value was less than 0.10. Analyses were implemented in R version 3.3.3, (R Foundation for Statistical Computing, Vienna, Austria).

## 3. Results

Cord blood mononuclear cell DNA was extracted in *n* = 162 samples. The MtDNAcn assay was successfully performed in *n* = 120 samples (median percentage of MtDNAcn: 68.4%, IQR 40.6–87.8) (Table 1). There were no statistically significant differences in participant characteristics by either infant sex or cookstove intervention cluster (Table 1).

### 3.1. Participant Characteristics

Participant characteristics are summarized in Table 1. Half of the infants were boys (*n* = 63, 53%). Wealth index was evenly distributed amongst the group. The majority of mothers had no (*n* = 43, 36%) or primary school only (*n* = 32, 27%) education. The median age of participating mothers was 25 years at enrollment (IQR 22–33) and the median gestational age at delivery was 39.7 (IQR 39.3–40.9).

Average prenatal CO exposure varied by arm (*p* = 0.02 by ANOVA), with participants randomized to the Control arm having a median average prenatal CO exposure of 1.07 ppm (IQR 0.75–1.60), BioLite arm median 1.15 ppm (IQR 0.53–1.41), and LPG arm 0.60 ppm (IQR 0.39–0.97).

### 3.2. Exposure-Response Associations between Average Prenatal CO and CBMC mtDNAcn

In the cohort as a whole, we did not identify a statistically significant association between increasing prenatal CO exposure and CBMC mtDNAcn (β = −2.15, SE = 2.68, *p* = 0.43 per 1 ppm increase in CO) following adjustment for maternal height and weight, ethnicity, age, and marital status; household wealth index; gestational age at delivery; child sex; and PCR plate number (Table 2).

Table 3 presents the sex-stratified analyses for the association between average prenatal CO and CBMC mtDNAcn. Among boys, increased prenatal CO exposure was associated with statistically significant reductions in CBMC mtDNAcn (β = −14.84, SE = 6.41, *p* = 0.03 per 1 ppm increase in average prenatal CO) following adjustment for maternal height and weight, ethnicity, age, and marital status; household wealth index; gestational age at delivery; child sex; and PCR plate number. No statistically significant association was seen in girls.

### 3.3. Effect of the LPG and BioLite Interventions on CBMC mtDNAcn

Median CBMC mtDNAcn in the LPG arm was 79.54% (IQR 63.23–98.53), BioLite 63.89% (IQR 50.85–78.39), and Control 58.30% (IQR 9.03–84.45).

Multivariable models identified a statistically significant association between cookstove intervention arm and CBMC mtDNAcn. Specifically, infants born to mothers randomized to the LPG arm had 19% higher relative CBMC mtDNAcn (β = 19.34, SE = 9.72, *p* = 0.049) compared to the 3-stone fire arm, following adjustment for maternal height and weight, ethnicity, age, and marital status; household wealth index; gestational age at delivery; child sex; and PCR plate number (Table 2). Infants born to mothers randomized to the BioLite Stove had no statistically significant difference in mtDNAcn as compared to 3-stone fire (β = 5.92, SE = 9.00, *p* = 0.51). When LPG was considered the referent, infants born to mothers randomized to the BioLite arm demonstrated a trend toward reduced CBMC mtDNAcn ((β = −18.67, SE = 10.51, *p* = 0.07), Table A1).

Sex-stratified analyses demonstrated that boys born to mothers randomized to the LPG stove had a 31% increase in mtDNAcn (β = 30.65, SE = 14.46, *p* = 0.04) as compared to 3-stone fire (Table 3). No difference in mtDNAcn was seen in boys born to mothers randomized to the BioLite arm (β = −7.75, SE = 11.63, *p* = 0.51) as compared to 3-stone fire. No statistically significant associations were seen in girls. P-interaction was not statistically significant (*p* > 0.10). When LPG was considered the referent, boys born to mothers randomized to the BioLite arm demonstrated reduced CBMC mtDNAcn ((β = −38.40, SE = 16.11, *p* = 0.02), Table A1) as compared to LPG.

### 3.4. Associations between CBMC mtDNAcn and Birth Outcomes

In the overall sample, multivariable linear regression models demonstrated statistically significant associations between CBMC mtDNAcn and both head circumference and gestational age at delivery. On average, a 1-percent increase in mtDNAcn was associated with a 0.013 cm increase in head circumference at birth (β = 0.013, SE = 0.06, *p* = 0.03) and 0.042 day increase in gestational age at birth (β = 0.042, SE = 0.019, *p* = 0.03) following adjustment for maternal height and weight, ethnicity, age, and marital status; household wealth index; gestational age at delivery; child sex; and PCR plate number (Table 4). While not powered for formal mediation, these findings suggest that infants born to the LPG arm would have a 0.25 cm increase (boys, a 0.40 cm increase) in head circumference and a 0.81-day increase (boys, a 1.29 day increased) in gestational age as compared to control.

## 4. Discussion

The objective of our study was to determine associations between prenatal CO, as a measure of HAP exposure, and CBMC mtDNAcn, a biomarker of mitochondrial function that reflects cumulative oxidative stress, and whether a prenatally-delivered clean cookstove intervention could reverse these findings. Exposure–response analyses suggest that boys are especially vulnerable to increases in prenatal CO exposure, with significant reductions in CBMC mtDNAcn. In all children an LPG clean cookstove intervention increased CBMC mtDNAcn as compared to both an improved combustion biomass stove (BioLite) and traditional 3-stone fire stove, suggesting that the cookstove intervention may reverse the effects of HAP exposure. Boys appear to especially benefit from the clean fuel intervention. Further, our data suggest that in all children increased CBMC mtDNAcn, such as that resulting from the clean cookstove intervention, is associated with improved birth outcomes including head circumference and gestational age.

The findings from previous studies on the relationship between prenatal air pollution and mtDNAcn in a variety of tissues are inconclusive. In cord blood, only one study has shown a significant and inverse relationship with prenatal ambient PM_2.5_ exposure, demonstrating an approximate 5% reduction in cord blood mtDNAcn per 10 μg/m^3^ increase in PM_2.5_ [23]; other studies have been negative. Associations between placental tissue mtDNAcn and prenatal ambient air pollution, including both ambient PM_10_ and NO_2_ exposures, have also been demonstrated [22,29]. Nonhuman primate models of prenatal exposure to tobacco smoke, which has many of the same harmful pollutants as HAP, have demonstrated a 25% reduction in mtDNAcn in aortic tissue [24]. To our knowledge, only one study has examined these associations in the context of adult exposure to HAP [30] and no study has examined the effects of prenatal HAP exposure or a prenatally-delivered cookstove intervention on CBMC mtDNAcn or birth outcomes.

In the current study, the clean cookstove intervention (LPG) arm had the lowest prenatal CO exposures. Further, this cookstove intervention was associated with increased CBMC mtDNAcn as compared to both the BioLite stove or 3-Stone Fire. These findings suggest that a clean cookstove intervention may be able to reverse the cumulative oxidative stress injury induced by prenatal HAP exposure. Oxidative stress secondary to increased air pollution exposure leads to an initial increase in mtDNAcn as a compensatory mechanism to increase the number of mitochondria in a cell in order to meet the cell’s energy demands. However, with continued or higher exposures, these compensatory mechanisms become overwhelmed resulting in a decreased synthesis of mtDNA [31]. This leads to a decrease in mitochondrial gene expression and ultimately, a decline in oxidative phosphorylation, cell senescence, and eventual cell death [32,33]. Across the lifecourse, mtDNA dysfunction caused by changes in mtDNAcn has been linked to adverse birth outcomes such as low birth weight, shorter birth length, poor infant growth, development of type II diabetes, cancer, and cardiovascular disease [22,34,35,36,37].

Our data suggest the effect of LPG cookstove intervention on mtDNAcn is not proportional to the effect of prenatal HAP exposure. In this GRAPHS subset, the median CO average prenatal exposure in the control arm was 1.07 ppm as compared to 0.60 ppm in the LPG arm, or a difference of 0.47 ppm between arms. Yet, among boys, the impact of the LPG cookstove on mtDNAcn was more than twice that seen with a 1 ppm increase in prenatal CO. It is possible that these differences are attributed to other air pollutants, such as PM_2.5_, that were not measured. Alternatively, it is plausible that other exposures, including other chemical [38] and nonchemical exposures, differed by cookstove intervention arm. A history of lifetime maternal trauma and psychosocial stress, for example, have been linked to placental and cord blood mtDNAcn and interactions between stress and air pollution exposures have been demonstrated [39,40].

Alterations in mtDNAcn with subsequent mtDNA dysfunction has been implicated in fetal programming thus changes in mtDNAcn may represent an intermediary pathway that links HAP exposure to adverse health outcomes [6,22,41]. Our data suggest that increased prenatal CO exposure or the use of a more polluting cookstove correlates with decreased mtDNAcn, which is further associated with reduced head circumference and gestational age at birth. Therefore, cumulative oxidative stress and the resulting impaired mitochondrial function may be one pathway mediating associations between increased prenatal HAP exposure and birth outcomes. This is consistent with previous findings by Clemente et al. in which NO_2_-induced changes in mtDNAcn were associated with decreased birthweight [22]. There, mediation analysis suggested that 10% of the association between prenatal NO_2_ and birthweight was due to changes in mtDNAcn [22]. Future studies of prenatal HAP would be strengthened by formal mediation analyses.

Sex-stratified analysis suggest that male infants are more sensitive to the effect of prenatal HAP exposure on CBMC mtDNAcn. A clean cookstove intervention may prevent these changes. These findings are in line with previous studies that similarly pointed to sex-specific differences in the effect of air pollution on cord blood mtDNAcn, with boys most affected [23]. Male fetuses are believed more vulnerable to oxidative stress [42] and more susceptible to environmental toxins [43]. Further, prior studies demonstrate sex-differential effects of prenatal exposures on a wide spectrum of child outcomes, including respiratory and neurocognitive outcomes [44,45]. While the mechanisms are not fully understood, the placenta is the maternal–fetal interface that buffers the developing fetus against maternal exposures. The placenta is sexually dimorphic and male and female fetuses have different placental transcriptomic profiles, thus may differentially respond to environmental toxicants [46]. Further, sex-differential structural and functional aspects, such as impaired invasion of uterine spiral arteries by male placentae and better surface differentiation of female placentae, may increase the vulnerability of male fetuses to environmental toxicants [46,47,48].

Our study demonstrates many strengths. This is the first study to exclusively randomize pregnant women to a cookstove intervention, including a clean-burning fuel stove, and perform extensive prenatal HAP exposure assessments. In a subset of children, cord blood was collected and CBMC mtDNAcn analyzed. We used the gold standard technique—duplex quantitative PCR—to measure mtDNAcn. Gestational age was determined at enrollment via ultrasound and birth anthropometrics measured within 24 hours of birth, allowing exploration of associations between mtDNAcn and birth outcomes. Our well-characterized cohort allowed for adjustment for a number of known confounders and covariates.

However, there are important limitations of note. While we adjusted for many important confounders, we did not have data for diet and other environmental exposures, both chemical and nonchemical, that may covary with air pollution. It is plausible that other environmental exposures, such as a history of maternal trauma, may have been unevenly distributed between study groups. Diet affects mitochondrial function [49] and dietary sources of antioxidants may modify the association between air pollution and mtDNAcn. Personal HAP exposure was measured at multiple time points, however due to logistics and cost we were unable to perform continuous personal monitoring throughout pregnancy. Finally, our sample size did not allow for formal mediation analyses, which would strengthen the clinical significance of our findings.

## 5. Conclusions

In conclusion, we have demonstrated that increased prenatal CO exposure is associated with reductions in CBMC mtDNAcn in male infants. Among all infants, but especially males, a prenatally-delivered LPG cookstove intervention to reduce HAP exposure attenuated these effects. Finally, CBMC mtDNAcn was associated with improved birth outcomes. Future studies are needed to confirm these findings and determine associations between CBMC mtDNAcn in disease processes across the lifecourse.

## Figures and Tables

**Table 1:** Participant characteristics.

Categorical Variables	All Children (*n* = 120)	%	Boys (*n* = 63)	%	Girls (*n* = 57)	%
Wealth index						
1 (Very poor)	27	22.5	15	23.8	12	21.1
2	26	21.7	12	19	14	24.6
3	22	18.3	11	17.5	11	19.3
4	18	15	9	14.3	9	15.8
5 (Least poor)	27	22.5	16	25.4	11	19.3
Maternal education						
None	43	35.8	20	31.7	23	40.4
1–6 years (Primary school)	32	26.7	21	33.3	11	19.3
>9 years (Secondary school)	45	37.5	22	34.9	23	40.4
Ethnicity						
Akan	22	18.3	10	15.9	12	21.1
Dagarti	34	28.3	18	28.6	16	28.1
Konkonba	19	15.8	7	11.1	12	21.1
Other (Gonja, Sisala, Mo, Fulani, Bimoba, Ga, or Banda)	45	37.5	28	44.4	17	29.8
Continuous variables (Median, IQR)	Median	IQR	Median	IQR	Median	IQR
Mitochondrial DNA copy number %	68.4	40.6–87.8	68.5	52.8–85.1	67.8	8.5–87.7
Maternal age (years)	25	22–33	24.5	21–32	28	23–33.3
Maternal height (cm)	155	151–160	155	150–160	155	152–160
Maternal weight (kg)	54.1	51–60	56	51–61.8	54	50–59.3
Gestational age at delivery (weeks)	39.7	39.3–40.9	39.8	39.3–40.7	40	39.3–41.2
Infant birth weight (kg)	3.02	2.71–3.21	3.03	2.79–3.21	3.02	2.61–3.22
Infant head circumference (cm)	33.9	32.4–35.0	34.0	32.4–33.5	33.5	32.3–35.0

**Table 2:** Association between prenatal carbon monoxide (CO) exposure and GRAPHS study arm, considered independently, on cord blood mononuclear cell mitochondrial DNA copy number (mtDNAcn) percentage *: Linear regression.

Exposure	*N*	Univariate Model		Multivariable Model †	
		β (SE)	*p*-Value	β (SE)	*p*-Value
Prenatal CO±	120	−1.78 (2.53)	0.48	−2.15 (2.68)	0.43
GRAPHS Study Arm					
3-Stone Fire	57	Referent		Referent	
BioLite Stove	35	9.61 (8.25)	0.25	5.92 (9.00)	0.51
LPG	28	20.75 (8.78)	0.02	19.34 (9.72)	0.049

* MtDNAcn percent change relative to the study population average. † Models adjusted for infant sex; gestational age at delivery; maternal height, weight, age, ethnicity; household wealth index; and PCR plate number. ± Per 1 ppm increase in average prenatal CO exposure.

**Table 3:** Sex-stratified associations between prenatal carbon monoxide (CO) exposure and GRAPHS study arm on cord blood mononuclear cell mitochondrial DNA copy number (mtDNAcn) percentage *: Linear regression †.

Exposure	*N*	Boys			*N*	Girls	
		β (SE)	*p*-Value			β (SE)	*p*-Value
Prenatal CO±	63	−14.84 (6.41)	0.03		57	0.67 (3.33)	0.84
GRAPHS Study Arm							
3-Stone Fire	30	Referent	NA		27	Referent	NA
BioLite Stove	17	−7.75 (11.63)	0.51		18	7.30 (15.37)	0.64
LPG	16	30.65 (14.46)	0.04		12	13.76 (17.63)	0.44

* MtDNAcn percent change relative to the study population average. † Models adjusted for gestational age at delivery; maternal height, weight, and age; ethnicity; household wealth index; and PCR plate number. ± Per 1 ppm increase in average prenatal CO exposure.

**Table 4:** Associations between mitochondrial DNA copy number (mtDNAcn) and birth outcomes: Linear regression *.

Birth Outcome	Univariate Model			Multivariable Model	
	β (SE)	*p*-Value		β (SE)	*p*-Value
Birth weight	0.001 (0.001)	0.20		0.0001 (0.001)	0.89
Head Circumference	0.014 (0.006)	0.02		0.013 (0.006)	0.03
Gestational Age at Delivery	0.034 (0.019)	0.07		0.042 (0.019)	0.03

* Models adjusted for infant sex, height, weight, and age; wealth index; ethnicity; and PCR plate number.

## Appendix A

**Table A1:** Association between GRAPHS study arm and cord blood mononuclear cell mitochondrial DNA copy number (mtDNAcn) *: Linear regression.

GRAPHS Study Arm	N	Multivariable Model †		Sex-stratified Analyses			
				Boys		Girls	
		β (SE)	*p*-Value	β (SE)	*p*-Value	β (SE)	*p*-Value
LPG	28	Referent	NA	Referent	NA	Referent	NA
BioLite Stove	35	−18.67 (10.51)	0.07	−38.40 (16.11)	0.02	−6.46 (20.16)	0.75
3-Stone Fire	57	−19.34 (9.72)	0.049	−30.65 (14.46)	0.04	−13.76 (17.63)	0.44

* MtDNAcn percent change relative to the study population average. † Models adjusted for infant sex; gestational age at delivery; maternal height, weight, and age; ethnicity; wealth index; and plate number.
